# Administrative Analysis of the Home-Based Older Persons Upstreaming Prevention Physical Therapy Program: A Pilot Observational Study

**DOI:** 10.7759/cureus.67290

**Published:** 2024-08-20

**Authors:** Christopher M Wilson, Sara K Arena, Kathryn Calleja, Shane Patterson, Hannah Randazzo

**Affiliations:** 1 Rehabilitation Services, Beaumont Health, Troy, USA; 2 Human Movement Science/Physical Therapy, Oakland University, Rochester, USA

**Keywords:** physical activity, exercise, gerontology, injury prevention, healthy aging, health coaching, geriatrics, rehabilitation, home safety, fall prevention

## Abstract

Introduction

Traditionally, physical therapist (PT) services do not commence until an injury, fall, or health issue has already occurred although there is increasing evidence that preventative programs administered by PTs may decrease the fall risk among elderly individuals. The purpose of this study was to examine billing, reimbursement, and administrative outcomes of the previously established and investigated prevention-based screening and intervention HOP-UP-PT (Home-based Older Persons Upstreaming Prevention-Physical Therapy) program delivered by a physical therapist in the home of older adults after being referred by a community partner. A randomized controlled trial of the HOP-UP-PT program demonstrated an 8-fold reduction in falls for participants at moderate and high fall risk compared to those who did not participate in the program.

Methods

A prospective observational study was performed to examine administrative and payment outcomes of HOP-UP-PT participation. Participants were referred into the HOP-UP-PT program via a local community center. Physician authorization for physical therapy participation was obtained for each participant as required for payment under United States' Center for Medicare and Medicaid Services (CMS) guidelines. The HOP-UP-PT program is preventative physical therapy delivered in the person's home with five in person visits (approximately one per month) followed by a monthly telehealth visit and a final in-person visit. Interventions included a balance program, home safety recommendations, health coaching, and addressing individual risks of falling or becoming homebound. A retrospective analysis was performed on the administrative and insurance payment data from this study which was then analyzed descriptively.

Results

Six participants with four different insurances completed the 7-month program (mean age=77 years) in 2021. The physical therapy visits were submitted to the participants' Medicare Part B plan. One participant's physical therapy visits were not submitted for payment as the health system did not have an active agreement with that health insurer. Due to the unclear status of telehealth visits in 2021, these services were not submitted to the insurance company for payment. All other PT visits were paid by the insurance companies. The mean amount paid for the initial evaluation code was $102.83 and the mean payment for the ~15 minute treatment codes was $25.90 per unit. Initial pilot data demonstrated a potential for a 4.2% positive operating margin when considering salary costs and travel. The mean delay from the initial referral into the HOP-UP-PT program until the physician provided written authorization for physical therapy was 69.7 days.

Conclusion

This study demonstrated initial evidence that payment for prevention-focused outpatient physical therapist services delivered in the home was feasible, however delays and costs in procuring physician authorization was a substantial barrier to prevention-focused physical therapy. A 4.2% operating margin demonstrated that, when efficiently operated, similar programs are likely to be viable. Furthermore, if telehealth services would have been paid, the operating margin was estimated to increase to 32%. Physical therapists are highly qualified to deliver efficient, effective preventative services which has the potential to reduce falls and institutionalization and subsequent healthcare cost savings.

## Introduction

The Centers for Disease Control and Prevention (CDC) reported that 14 million (27.6%) older adults reported falling in 2019 [1]. Additionally, the annual cost for treatment related to falls in the United States' Medicare program is $31.3 billion [2]. When an older adult is no longer able to safely live at home (e.g., extended care facility, or assisted living), the costs to the older adult, the health system, and society are expected to increase [2]. With the anticipated population growth of older adults in the coming years, there is also an expectation that the annual cost to Medicare and other insurance payers will increase.

Traditionally, physical therapist (PT) services do not commence until an injury, fall, or health issue has already occurred although there is increasing evidence that preventative programs administered by PTs may decrease the fall risk among elderly individuals [3]. Reducing a person's fall risk may, in turn, decrease the need for hospitalization, institutionalization, and unwarranted medical and surgical procedures, thereby reducing the financial burden on the healthcare system. In order to appreciate the benefits of such programs, there needs to be widespread operationalization in order to “identify older adults at risk for becoming homebound, experiencing a functional decline, or falling” [4]. The Home-based Older Persons Upstreaming Prevention Physical Therapy (HOP-UP-PT) program is one such program that has evidence that it can decrease falls and fall risk indicators among older adults using a preventative, multimodal, and comprehensive approach [3-6]. HOP-UP-PT is an established preventative care program that enables community partners such as senior centers to identify older adults who may be at risk of falling or becoming homebound and refer them to a PT. The PT then provides a comprehensive geriatric assessment to the older adult including assessing their home environment, balance, strength, and cognitive status [7,8]. If the older adult qualifies for the program, a plan of care is established, and interventions occur over a seven month time frame where the PT provides six in-person and three telehealth visits in the home of the older adult. Although the program is prevention-focused and has been administered successfully and safely in prior study phases, some insurances, such as Medicare, require a signature from a physician or non-physician practitioner (physician's assistant, clinical nurse specialist, or a nurse practitioner) on the plan of care or prescription for outpatient PT services [9].

A randomized controlled trial demonstrated an eight-fold reduction in falls for those with moderate- and high-fall risk who participated in the HOP-UP-PT program (n=72) as compared to those who did not participate in the study [3]. Additionally, all aspects of the screening, participant assessment, and interventions are within the scope of routine PT services; however, the referral pattern (from a community center) and service delivery location (in the home of the individual who does not meet the Medicare definition of homebound) is a novel service delivery model and reimbursement amounts and administrative costs and burdens related to these services are not known.

A follow-up study was conducted to determine the intermediate-term outcomes of the HOP-UP-PT program via telephone follow-up with participants at three-month and six-month checkpoints [6]. Adults over the age of 65 years reported annual fall rates of 28.8-39.9% [1,10]. It was determined from post-program questionnaires at both checkpoints (n=77, 71) that the rate of fall occurrences was only 16% [6], further supporting the efficacy of the HOP-UP-PT program. Additionally, the participants had an overall positive perception of the program and the resultant outcomes that they had experienced [6]. As it relates to the financial feasibility of the program, many of the participants stated that they would be willing to pay for a portion of the services provided but the average amount that the participants expressed that they would cover was around 10% of the actual program costs [6]. In the presence of this significant gap between the actual costs of the program and what the participants are willing to pay, finding another avenue to cover the cost of HOP-UP-PT is warranted to support the financial viability of the program.

The average cost of an unintentional fall that required medical attention in 2021 was estimated to be $37,436 [11]. Utilizing this data, a fall prevention-focused program like HOP-UP-PT, implemented in a financially sustainable manner, may have a significant impact on cost-savings for the medical system. Extrapolating the findings of the randomized controlled trial, performed in 2021 by Arena et al., to 100 older adults with elevated fall risk who would participate in the HOP-UP-PT program, the reduction in falls could result in a savings range of $123,500 to $1.6 million [3]. In a recent report from the American Physical Therapy Association (APTA), 'The Economic Value of Physical Therapy in the United States,' the benefit of fall prevention was analyzed through quality-of-life measurements. It was found that physical therapy-based falls-prevention services led to an average estimated net benefit of $2,144 per episode of care [12]. With the significant financial burden placed on the healthcare system from the growing number of falls, there is substantial evidence that cost-saving prevention programs such as HOP-UP-PT warrant payment through public and private insurers in order to decrease this growing cost risk.

The purpose of this study was to examine billing, reimbursement, and administrative outcomes of the previously established and investigated prevention-based screening and intervention program carried out by a PT in the home of older adults after being referred by a community partner. Despite evidence showing the efficacy of HOP-UP-PT, it was not clear that conventional outpatient insurance payment would be sufficient to cover the therapist’s salary, overhead (time/cost associated with travel, a scheduler or biller’s salary), or mileage. This is an anticipated barrier to integrating preventative programs such as HOP-UP-PT into the standard of practice and the realization of reduced costs of falls and institutionalization [3].

## Materials and methods

Study design

This was a prospective observational study of the billing, payment, and administrative outcomes of a pilot implementation of conventional outpatient billing of the HOP-UP-PT program utilizing a variety of Medicare and Medicare Advantage insurance plans. Medicare is the United States' federal health insurance for individuals over 65 as well as some individuals younger than 65 with certain disabilities or conditions and outpatient procedures are covered under Medicare Part B. Instead of conventional Medicare coverage, individuals can elect to enroll in Medicare Advantage plans that offer different coverage options and are administered by private companies. Finally, some individuals also elect to have a secondary health insurance that may provide coverage for items that are not covered under the patient's primary insurance or covers items like copays or deductibles.

This study was approved by the Henry Ford Health System’s (Detroit, MI, USA) Institutional Review Board (IRB # 13704; approval date 6 May 2020) and the Oakland University (Rochester, MI, USA) Institutional Review Board (FY2020-199, approval date 19 June 2020). The rights and privacy of participants were protected at all times. For each in-person visit, each participant received a $35 research incentive for their participation. The health system was also provided with payment from grant funding for the provision of the professional and administrative services. 

Procedures

All study procedures occurred in one local suburban community near the city of Detroit, MI, USA. Despite the HOP-UP-PT program being previously available in other communities in Michigan, this was the first time this program was offered in this community. Recruitment of participants began in February 2021, with the local community center providing advertisement for the study and then notifying the primary investigator of a potential participant. All participants were initially contacted by phone to explain the study procedures between February and March 2021, with the recruitment period ending in March.

The HOP-UP-PT program takes place in the home of the older adult. Over the span of seven months, the program included six in-person visits and three virtual telehealth visits with program procedures previously described by Wilson et al [5]. As the participants were not homebound, their personal health insurance was billed for each in-person visit as an ambulatory/outpatient procedure (e.g., Medicare Part B or Medicare Advantage plan) and participants were responsible for any co-pays or deductibles as their insurance coverage policy required. As coverage for telehealth visits remained uncertain at the time of the study and may not have been consistently covered in various health plans, the telehealth visits were not submitted for payment. 

Participants

Study participants were included if they were greater than or equal to 65 years of age, if the community center staff subjectively identified them as ‘at risk’ for decline in community-dwelling status, were willing to participate in a home-based program provided by a licensed PT, and had Medicare or Medicare Advantage as their primary health care insurance carrier. Participants were not required to have a secondary health insurance.

Participants were excluded from the study if they met any of the following criteria: received physical therapy services within the past two months in any setting, been admitted to a hospital within the prior two months, were receiving palliative or hospice care at the time of the program, or if the initial evaluation by the licensed PT suggested medical or cognitive status would not permit safe program participation without further medical evaluation. Additionally, participants were not included if they had a health insurance provider outside those provided in the inclusion criteria as the HOP-UP-PT is focused on older adults, and most older adults in the US are covered by Medicare or a Medicare Advantage plan. 

Data collection

The variables selected for analysis included three main categories: billing, payment, and administrative outcomes. Due to the wide variety and nature of the data, multiple sources of data were utilized including retrospective analysis of each participant’s electronic medical record, billing reports, and tracking logs developed by the investigators to monitor clinical and administrative milestones (e.g., length of time between initial participant contact to physician authorization to beginning and ending the program). 

For billing outcomes, data points included the type and number of units billed including the average units billed per common procedural terminology (CPT) code and visit, the average dollar amount billed for each CPT code by visit, and the average dollar amount that insurance paid per visit (CPT codes are a registered trademark of and the property of the American Medical Association). As a part of many insurance company contracts, healthcare providers only receive a percentage of the amount of money that is billed to the insurance - this is termed the allowed amount. Billing data was provided by the health system’s home healthcare division. From this information, the researchers calculated an average reimbursement percentage amount, which would be determined through a comparison of the ratio of the average amount billed per visit to the average allowed amount paid per visit. Another variable examined was the units billed versus the PT’s total time spent with the patient, as the start and end time for each PT visit was tracked utilizing either an electronic app (Rover, EPIC LLC, Madison, WI, USA) or via retrospective analysis of the electronic documentation.

For operational outcomes, the researchers calculated if the insurance payment received was sufficient to cover salary and overhead costs for care delivery, also known as a break-even point (expenditures equivalent to income). The final variables examined included the amount of time for telehealth visits and patient out-of-pocket costs.

The administrative outcomes analyzed were as follows: time and salary costs to train and prepare a clinical site/PT to carry out the HOP-UP-PT program, overhead and time costs of obtaining written authorization for physical therapy from each participant’s primary care physician (PCP) and setting up the initial visit, and average time for an initial evaluation, daily treatment, and telehealth visit. Each data collector PT received a one-time four-hour training session. Although not a requirement within the state of Michigan, a signed authorization was obtained as a requirement of most private insurance and Medicare for PT services. This data was analyzed to approximate labor, financial costs, and length of delays for the physician clearance for this prevention program. Finally, the cost required for a participant to see their physician before beginning the HOP-UP-PT program (if necessary) was estimated based on publicly available data and resources. As prior phases of the HOP-UP-PT program did not require the added time and cost of a physician visit or obtaining a signed authorization for this low-risk prevention program, this data was important to examine to determine the extent of cost and time to the patient, the healthcare system, and the patient's insurance company.

Data analysis

All data was analyzed utilizing descriptive statistics. Measures of central tendency and standard deviations were calculated to assess trends based on the distribution of the data. Due to the small sample size and the nature of the data, inferential statistics were not appropriate.

## Results

All participants were referred through one local community center which serves the metropolitan region of Detroit, MI, USA. Eight total participants were referred to the HOP-UP-PT program; however, two declined enrollment. The demographics of the enrollees (n=6) were as follows: mean age = 77 years old (standard deviation=5.67; range=70-86), two male and four female participants; one African American and five Caucasian participants. In order to preserve the anonymity of the participants and the insurance providers' proprietary plans, the commercial Medicare Advantage plans were coded as MA Plan A (n=2), MA Plan B (n=1), and MA Plan C (n=1), while two people had conventional Medicare with a commercial secondary insurance provider (MC+SI).

No billing data on the participant with MA Plan C (Participant 6), was obtained as the health system’s homecare agency did not have an executed provider contract for outpatient services. Retrospective attempts to obtain payment for these services were not attempted.

Financial outcomes

Billing Outcomes

Among the other five participants, the average amount billed per visit was $323.67. In contrast, the average amount paid by insurance per visit was $97.23. In the analysis of billing and payment, six visits were analyzed instead of nine because the three telehealth visits were not billed for any of the participants, leading to a total of 30 billable visits among the five participants (Table 1). A visit was defined as each separate date of service. For Participants 1, 3, 4, and 5, this meant that the initial evaluation and first treatment episode occurred on the same date of service, Visit 1 (although billed as at least two separate CPT codes: an untimed evaluation code, plus a timed treatment code). For Participant 2, Visit 1 consisted of only an evaluation (an untimed evaluation code).

**Table 1:** Types of Billing Codes and Payment Received CPT = Common Procedural Terminology. CPT® Codes are the property and registered trademark of the American Medical Association.

CPT Code Billed Among Participants	Frequency of Each CPT Code Billed	Average Amount Received per CPT Code	Total Amount Received by CPT Codes
97161 (Low Complexity Evaluation)	4	$102.83	$411.32
97162 (Moderate Complexity Evaluation)	1	$102.83	$102.83
97110 (Therapeutic Exercise)	91	$25.90	$2,357.23
97140 (Manual Therapy)	3	$22.22	$66.66

Across six visits, the average number of units billed per visit, including the evaluation unit, for all five patients was 3.30 (Table 2). The average units billed excluding the evaluation unit and telehealth units (PT treatments only) was 3.26. The average time for a PT non-virtual visit, excluding the first visit for participants one, three, four, and five due to the fact that the evaluation time and treatment time were combined, was calculated to be 50.1 minutes. The researchers then compared this number with the average units billed per PT visit, excluding the untimed evaluation unit (3.26). The number of units billed appears to be in alignment with the guidance provided by the Medicare 8-minute rule, which states that PT visits between 38-53 minutes should result in billing 3 units. The Medicare 8-minute rule is guidance provided by Medicare to determine the number of CPT units that is appropriate to bill Medicare for physical therapy services [13].

**Table 2:** Number of CPT Units Billed per Visit Visit 1, 5, 9 = Initial in-home evaluation and re-evaluation visits (including comprehensive assessment of tests and measures); Visit 2-4 = In home treatments; Visit 6-8 = Virtual telehealth visits. CPT = Common Procedural Terminology. CPT® Codes are the property and registered trademark of the American Medical Association.

	Visit 1	Visit 2	Visit 3	Visit 4	Visit 5	Visit 6 (telehealth)	Visit 7 (telehealth)	Visit 8 (telehealth)	Visit 9
Mean CPT Codes Billed Per Visit	4	2.8	2.2	2.6	4.4	N/A	N/A	N/A	4.4

Using the time-in/time-out data, the total number of minutes for each PT visit was calculated. The researchers then compared these times and units billed with the Medicare 8-minute rule. When analyzed, there were six circumstances where there was evidence of billing deviations from the Medicare 8-minute rule, four of which were billing one less CPT unit than the allowable amount (negative deviation of -$107.40) and two of which occurred where one additional CPT unit was billed beyond the Medicare 8-minute rule guidance (positive deviation of $51.76). Overall, the therapist mostly billed appropriately with only an estimated total loss of $55.64 among all 30 visits. These estimates were created under the assumption that the therapist accurately used the Rover app to capture all minutes with the patient. While it is possible that errors can occur due to technical failures or user errors, none were identified during this study as the health systems' quality assurance process included comparing the time inputted by Rover with the data collector PT's clinical note.

Payment Outcomes

The average amount paid by insurance per visit to the health system after contractual adjustment was $97.23. As a percentage, the average amount paid amongst all participants’ insurance was 30.0% of the average amount billed (Table 3).

**Table 3:** Amount Billed and Payment Received Per Visit MA=Medicare Advantage; MC+SI=Medicare and Secondary Insurance

Participant Identifier	Primary Insurance	Number of Visits	Mean Units Billed per Visit (Including Evaluation Code)	Mean Units Billed/Visit (Excluding Evaluation Code)	Mean Amount Billed per Visit (Including Evaluation Code)	Mean Payment per /Visit (excluding Evaluation Code)	Mean Percentage of Payment Received vs Billed
1	MA Plan A	6	3.50	3.33	$343.33	$102.24	29.8%
2	MA Plan A	6	2.83	3.20	$278.33	$86.38	31.0%
3	MC+SI	6	3.50	3.33	$342.50	$102.07	29.8%
4	MA Plan B	6	3.33	3.17	$327.50	$98.11	30.0%
5	MC+SI	6	3.33	3.25	$326.67	$97.35	29.8%
Total		30	3.30	3.26	$323.67	$97.23	30.0%

Operational outcomes

For each participant, the days between the initial contact with the participant through the final visit with each participant are shown below (Table 4).

**Table 4:** Time Between Key Events for the HOP-UP-PT Program HOP-UP-PT=Home-based Older Persons Upstreaming Prevention Physical Therapy

Participants	Days from initial contact with physician to when the physician order/authorization was received	Days from when physician order/authorization was received to when first visit occurred	Days from first visit to ninth visit
1	74	3	214
2	29	11	253
3	29	19	245
4	67	2	222
5	71	19	236
6	148	25	216
Mean (Standard Deviation)	69.7 (43.5)	13.2 (9.4)	231 (14.8)

Prior to initiating the HOP-UP-PT program, the primary care physicians (PCPs) of two of the five participants requested to see their patients prior to their enrollment in HOP-UP-PT. Furthermore, one participant was required to establish a new physician relationship which included an in-person visit prior to initiating the HOP-UP-PT program. After the visit, the referral to the HOP-UP-PT program was delayed due to a necessary medical follow-up and surgical procedure. The subject was then cleared to participate 54 days after the original HOP-UP-PT referral was initiated from the community center. 

As direct costs and payment for the physician office visits were not readily available to researchers, a proxy measure to estimate physician office visit costs was determined. The Center for Medicare and Medicaid Services (CMS) Physician Fee Schedule Search Tool was utilized as it includes a comprehensive list of fee maximums used to reimburse physicians and other healthcare service providers for covered services based on geographic location [14]. This tool can be accessed at https://www.cms.gov/medicare/physician-fee-schedule/search. In order to estimate the cost of a standard PCP visit, the Fee Schedule tool was used to assess MAC locality 0820201: Detroit (which includes Macomb, Oakland, Wayne, and Washtenaw counties) for the year 2021. The investigators then used an online medical code referencing tool, Codify, to determine common physician billing codes. Codify provides an index of CPT codes defined by certain medical procedures or services [15]. Two CPT codes were then chosen after using Codify to identify the likely type of office visit billed for the subjects, CPT 99214 and 99204. CPT 99214 is an established patient office visit involving the evaluation and management with moderate decision-making and/or 30-39 minutes of encounter on a single date. Costs for this CPT code are recommended by CMS to range from $134.06 to $146.46 for non-facility (office) visits or $103.45 to $113.01 for facility (outpatient) visits. CPT 99204 is a new patient office visit involving evaluation and management with moderate decision-making and/or 45-59 minutes of encounter on a single date. Costs for this CPT code are recommended by CMS to range from $174.71 to $190.87 for non-facility (office) visits or $142.35 to $155.52 for facility (outpatient) visits. 

Each cost per CPT was averaged, to find the following mean costs: 99214 (non-facility): $140.26, 99214 (facility): $108.23, 99204 (non-facility): $182.79, 992014 (facility): $148.94. According to Medicare.gov, for Medicare Part B an insured person “typically pays 20% of the Medicare-Approved Amount for most doctor services” after the deductible of $233 is met (https://www.medicare.gov/basics/costs/medicare-costs) [16]. If the patient has not met their deductible, they could be responsible for paying a partial or full amount of the above amounts billed. If the patient has met their deductible, the mean estimated out-of-pocket cost for the PCP visit to be $28.05 (non-facility, existing patient), $21.65 (facility, existing patient), $36.56 (non-facility, new patient), and $29.79 (facility, new patient), which is 20% of the mean amounts aforementioned.

*Operating Margin Analysis*

Based on our financial analysis, the insurance payment was projected to cover the estimated salary and overhead expenses of the six in-person and three telehealth visits by a homecare PT. We estimated that the PT’s salary at the time of data collection for each of the six in-person visits was $70 and $35 for each of the three telehealth visits. These salary estimates include fringe costs (e.g., benefits) for the clinician as well. With these values in mind, the average salary per visit, across nine total visits, was $58.33. For our overhead cost, we calculated the travel expenses using the 2021 federal reimbursement amount of $0.58 cents per mile and multiplied that by an estimated average distance traveled by the PT of 10 miles per visit, roundtrip. When calculated, the travel cost per visit would equal $3.87. The combined cost of the average salary of $58.33 and the average travel cost of $3.87 equaled a total of $62.20.

The average amount reimbursed per visit for all nine visits (inclusive of the unbilled telehealth visits) was $64.82 per visit. This average exceeded the salary and overhead costs of $62.20 by $2.62, a positive operating margin of 4.2%. Only one participant’s case did not exceed $60.26 as they had an average allowed amount of $57.59. This may be due, as previously mentioned, that the PT underbilled for the time spent with this patient.

A similar breakdown was done to assess the cost difference if a physical therapist assistant (PTA) had been the one giving the treatment for the last four in-person visits and all three telehealth visits. This was found using an estimated salary of $60 per in-person visit for a PTA and $30 per telehealth visit.

The average salary across the nine visits with a PT providing the interventions for the first two visits and the PTA treating for the remaining seven visits was $52.22, compared to $58.33 per visit when all nine visits are provided by a PT. In regard to payment, PTAs are paid at 85% of the usual payment rate of a PT utilizing the Medicare Fee Schedule [17]. To obtain the average reimbursement rate across nine visits for a PTA to administer treatment, we multiplied the previously established allowed amount by 0.85, which resulted in a value of $58.97, resulting in a positive operating dividend of $2.88 per visit (positive operating margin of 4.8%) when a PTA administers treatment for the last seven visits of the program. 

The overhead cost for nine visits (this includes the three telehealth visits) as delivered by a PT was determined to be $559.80 (inclusive of salary and travel cost). The average number of CPT code units that would be required to be billed per visit (excluding the evaluation code) was 2.94 units in order to break even. The same methodology was used to calculate the break-even point when the last seven visits (including the three telehealth visits) were provided by a PTA where the overhead cost would be $504.80, requiring 2.87 units per visit to break even (excluding the evaluation code). These break-even points were established without the presumption of payment for the telehealth visits.

Administrative outcomes

*Initial Startup Costs* 

In order to prepare a provider to deliver the HOP-UP-PT program, the PTs must receive adequate training, which includes a four-hour initial training course followed by three to four meetings with home care administrators which require approximately one hour each. The training costs for one PT to be trained in program delivery were $140 ($35/hour for participating in meetings) and approximately $105-$140 for the three to four visits with the administrators of the home healthcare agency. In addition to the direct salary costs of training and meetings, the unrealized income from patient care provision during the PT training was approximately $414 ($25.90 for 1 unit of 97110 x 16 units). 

Indirect Costs

For the implementation of this program, this home healthcare provider did not have previously established billing processes for Medicare Part B so this required development before the program could begin. It took a series of meetings to build a billing and documentation portal within the institution’s electronic medical record which was not able to be directly quantified.

Unrealized Income for Telehealth Visits

In 2021, the 97000 series CPT codes were not consistently able to be billed when delivered via telehealth. Additionally, some payers required an addendum attached to the bill that specifies that the services were provided via telehealth and with an explanation of the charges or a code modifier delineating services were delivered via telehealth [18]. For example, Modifier 95 indicates that synchronous telehealth services were rendered via real-time interactive audio and video telecommunication systems; or a Modifier of Place of Service (POS) 10 would indicate that telehealth services were rendered in a patient’s home [19].

To calculate the lost income for telehealth visits, the researchers made the assumption that the PT would have received contemporary payment for every telehealth service provided throughout the study for the five participants who received reimbursement. The PT spent an average of 31.0 minutes per telehealth visit with the five participants; every participant had six total units across all three telehealth visits that could have been billed. In total, 30 units were not able to be billed. Since all but one in-person visit (excluding initial evaluations) were billed as 97110 Therapeutic Exercise, the researchers anticipated that the telehealth visit would also be billed as this CPT code. Based on this assumption, the expected total reimbursement amount for all telehealth visits across all five participants would have been $777. As there is no travel involved for the telehealth visits, the overhead costs would equate solely to the salary of the PT. The therapists were paid $35 dollars per telehealth visit, so the total salary cost across the total telehealth visits was $525. Using this value, it is predicted that the additional income for the five patients, had telehealth been billed for, would have been $252.

Delays and Overhead Costs

Delays in getting the written physician authorization (i.e., initial referral and/or signed plan of care) signed present a potential loss of payment or delay in payment if the authorization is delayed significantly or is never signed. Most insurance companies have a time limit in which procedures can be billed for payment and if the time to procure the physician authorization exceeds the time limit, the administered procedures will not be paid, thereby resulting in a loss of income for services rendered. In addition to potential payment delays or losses, there may also be increased administrative costs to procure approval for a signed authorization (e.g., administrative salary for tracking, calling, and follow-up), which was not able to be directly tracked, but it is estimated that procuring written authorizations may result in additional costs of $20-$40 per participant.

There was a significant delay in payment for two of the visits for one participant who had Medicare as their primary insurance and Blue Cross Blue Shield as their secondary insurance. Additionally, there were two virtual and one in-person visits for this subject that occurred in the first three months of 2022. Because it was a new calendar year, the patient’s insurance deductible reset and the patient had to pay $101.30 out-of-pocket for this visit.

## Discussion

When appropriately billed, an outpatient visit provided by a homecare PT for a prevention program demonstrated a net revenue of $2.62 per visit which equated to a 4.2% operating margin, despite not billing for the telehealth visits. Additionally, it was estimated that a potential model for the program that includes the services of a PTA would only increase the operating margin to 4.8%. As a result of this small increase, the researchers believe that an attempt to implement the PTA-involved model of care would be revenue-neutral however from a manpower standpoint, using PTAs for these treatment visits may allow PTs to instead be available for initial evaluations or more complex patient care procedures. The 4.2% operating margin provides evidence that as long as extraneous variables (e.g., excessive travel mileage or underbilling) are controlled, the HOP-UP-PT program has early evidence of fiscal viability; however, due to training and building the administrative infrastructure, a delay in a positive operating margin can be anticipated. This potential delay would be defrayed over time by the volume of visits. 

The fiscal viability of the program could be further supported through the billing of the rendered telehealth services. Due to constantly evolving billing regulations (particularly for telehealth) reimbursement for telehealth services is hard to predict [20]. Despite this unpredictability, if it were assumed that there was full reimbursement for the telehealth visits that were rendered in this study (a value determined earlier to be $777), then the operating margin would increase by seven-fold up to 32% for the program as opposed to the previously mentioned 4.2%. The total payment would increase to $3,693.86. This is in contrast to the total overhead cost of the study of $2,799. Even if only a fraction of the unbilled telehealth units were paid, the potential increase in the operating margin may still be significant. With this in mind, establishing the best current practice of billing for telehealth visits for a patient in the home setting to maximize reimbursement is of the utmost importance to support the sustainability of the program [21]. 

There were significant delays in receiving the signed physician authorization for two participants. The PCPs of two of the participants insisted that the participants have an office visit before enrolling in this preventative fall prevention program. Additionally, one participant had to establish a new patient visit with a new physician prior to enrolling. For this particular subject, there was a further delay due to needing a medical follow-up and surgical procedure. This delayed enrollment by 54 days after initiating the referral. Although this is a small sample size, out of the six participants, 50% required additional physician services, which incurred additional costs on the payor and likely the participants themselves. In future studies and pilot programs, the PT should account for these delays, as participants may not be medically stable to participate in exercise upon initial contact with physicians. Delays in physical therapy due to the need for physician authorization prior to beginning PT care resulting in increased costs and disability have been previously established in the literature [22,23].

Limitations and future research

This study is limited in its generalizability, as the researchers were only able to gather data on three unique insurance scenarios - two with primary Medicare and a commercial secondary insurance and three different Medicare Advantage plans. Each insurance company is unique in its reimbursement and coverage, which provides a challenge for establishing uniform payment and access to preventative programming. A larger sample size is required to gain a better understanding of the operating margin totals and administrative costs. Although limited, this study serves as an exploratory foundation for a larger, more comprehensive analysis of insurance payment for PT-delivered preventative services in the home. 

This study had one Medicare Advantage insurance plan, MA Plan C, for which an outpatient provider contract was not fully executed which illustrates another likely barrier for home healthcare agencies to provide this service, procuring outpatient insurance contracts. A substantial administrative barrier for this home healthcare agency was developing new processes to document and bill for outpatient procedures; it may be more advantageous for home healthcare organizations to partner with their outpatient counterparts to utilize their processes for documentation and billing, if applicable. In the future, the program should anticipate inconsistent payment methods for preventative care or telehealth services delivered by PT services; and some participants may decline preventative care as there is a risk of out-of-pocket costs for services when payment is denied. With a positive operating margin found, as well as previously found cost-savings range of $123,500 to $1.6 million, providers of prevention-focused PT services may consider collaborating with a wide variety of insurance companies in the future to establish consistent, sustainable payment methods for their preventative services [3]. 

## Conclusions

A prevention-focused multimodal program provided by PTs in older adults’ homes demonstrated that when billed, these services were paid for by several common insurance providers. A positive operating margin of 4.2% was identified with a potential for a 32% positive operating margin if a consistent pathway for telehealth payment is established. Furthermore, it was determined that to reach a break-even point for the HOP-UP-PT program, the PT would need to bill at least three units per visit. Despite the fact that prior studies of prevention-focused programming delivered by PTs have been shown to be safe and effective without prior physician authorization, there were substantial delays in procuring a signature on a plan of care to initiate this program. These delays could deter patients from receiving these services or seeking healthcare services from unlicensed, unqualified providers for which a referral or a signed plan of care is not necessary. These delays could also increase the risk of falls and downstream healthcare costs. In order to fully leverage the effectiveness of programs similar to HOP-UP-PT, efforts should be focused on reducing unwarranted administrative burden and out-of-pocket costs to the patient in order to optimally appreciate the health benefits and reduced healthcare utilization costs.
